# A review on phytochemical and bacteriophage based therapeutic strategies for the management of Brucellosis

**DOI:** 10.3389/fvets.2026.1727080

**Published:** 2026-05-01

**Authors:** Rajshree Vidyashankar, Subhash Verma

**Affiliations:** Department of Veterinary Microbiology, Dr G C Negi College of Veterinary and Animal Sciences, CSK Himachal Pradesh Agricultural University, Palampur, Himachal Pradesh, India

**Keywords:** antimicrobial resistance, bacteriophage, botanicals, Brucella spp., innate immunity, phage engineering

## Abstract

Brucellosis, a zoonotic disease, triggers severe inflammation and reproductive complications in both humans and animals. The standard treatment protocol relies on prolonged antibiotic courses, often combining streptomycin and doxycycline, or gentamicin and doxycycline, for a duration of six to eight weeks. However, concerns have arisen regarding the efficacy, with a notable 15% failure rate and frequent relapses. The lack of suitable vaccines and the rise of antimicrobial resistance in *Brucella* spp., due to prolonged antibiotic use further complicate the issue. This review underscores the pressing issue of antibiotic resistance, prompting an exploration of alternative treatment avenues. Phytochemicals like polyphenols and terpenoid rich essential oils have been reported to possess strong anti-Brucellar activity and function by disrupting cell wall and membrane functions, prevent binding to host cells and internalization and inhibit DNA replication and transcription and enzyme synthesis. Similarly, bacteriophages, have demonstrated promise as diagnostic, therapeutic and biocontrol agents in Brucellosis management. The efficacy of bacteriophage mediated strategies can be enhanced by site specific delivery through nano-encapsulation techniques while engineered phages can be used for diagnosis and modulate immune response in host. These alternative therapies have even been found to stimulate innate immunity in host. However, the precise mechanisms through which these treatments operate remain elusive, warranting further investigation. In conclusion, this paper advocates for the integration of novel plant-based therapy as adjuvant treatment option for both human and livestock brucellosis, while phage therapy as complementary strategy to augment the conventional treatments in livestock brucellosis. These non-antibiotic alternatives aim to improve treatment efficacy, reduce the required duration of conventional antibiotic regimens, and minimize economic losses in agriculture. The future of brucellosis management may lie in a multi-faceted approach that combines antibiotics with innovative, natural alternatives.

## 1 Introduction

Brucellosis is a zoonotic disease primarily affecting livestock, caused by a Gram-negative coccobacillus called *Brucella* spp., mainly *Brucella abortus*, and occasionally by *Brucella melitensis* and *Brucella suis* (1). Livestock, particularly bovine species, can get infected through various means such as insemination by an infected bull, close contact with an infected animal, or ingestion of contaminated water. *Brucella* infection in bovines can lead to abortion in the last trimester, with the aborted fetus, placenta, and uterine secretions serving as sources of infection for other animals in the herd (2). The disease can be transmitted to humans through direct contact with infected animals or consumption of contaminated livestock products (3). There are 12 known species of *Brucella*, with *B. melitensis, B. abortus, B. suis*, and *B. canis* reported to cause disease in humans, with *B. melitensis* being the most virulent (1). Brucellosis results in significant economic losses globally due to reduced animal health and productivity. For instance, Singh et al. (4) estimated that the annual median economic loss due to Brucellosis in India is nearly USD 3.4 billion, with cattle accounting for approximately 96% of infections. This is a major concern given that India is the world's largest milk producer. The global incidence of human brucellosis is conservatively estimated at 2.1 million cases annually, making it a significant global health issue, particularly in resource-limited countries in Africa, the Middle East, Southeast Asia, and South America (2, 5).

*Brucella* spp. are facultative intracellular bacteria that infect by exploiting the host's immune defenses and typically cause chronic infections (6–8). *Brucella* infections exhibit a wide range of clinical manifestations in humans, including fever, fatigue, and joint pains. While Brucellosis is rarely fatal in humans, long-term persistence of the infection can lead to severe disabilities such as endocarditis, neurological disorders, and, in some cases, death. *Brucella* infection was previously known by various names such as Malta fever, Mediterranean fever, Gibraltar fever, Cyprus fever, and undulant fever (9). Diagnosing brucellosis involves bacteriological, molecular, and *in vitro* serological methods. The bacteriological method, which involves cultivating *Brucella* spp., from infected tissue samples or fluids, is considered the “gold standard” (1). Effective control strategies for Brucellosis in livestock include frequent surveillance, prevention of transmission, controlling the reservoir of infection, and vaccination (2). For human brucellosis, the primary treatment option is a prolonged period of antibiotic supplementation. Combination therapy with two or more antibiotics is often preferred for treating brucellosis (10). Despite the effectiveness of antibiotic treatment in controlling the infection and symptoms of brucellosis, relapses have been observed in some cases. The relapse rate in uncomplicated cases of Brucellosis ranges between 5% and 15% (10). This can be attributed to the intracellular location of the bacteria, making antibiotics less effective in acidic phagolysosomal environments and requiring prolonged antibiotic supplementation (11). However, prolonged antibiotic treatments pose the risk of antimicrobial resistance (AMR) and associated morbidities. AMR is a global threat to human health, causing nearly 4.95 million deaths (12). Studies have shown AMR against trimethoprim-sulfamethoxazole and azithromycin in *B. melitensis* isolated from infected patients (13). Reports of mild resistance against rifampicin, ampicillin-sulbactam, and colistin have also been observed in some countries (3, 14). In countries where Brucellosis is endemic, other infectious diseases like tuberculosis, which require prolonged antibiotic treatment, coexist. This coexistence increases the likelihood of AMR against commonly used antibiotics (11). Therefore, it is crucial to identify alternative therapies for combating Brucellosis.

Alternative therapies effective for treating and controlling Brucellosis include vaccination for livestock, supplementation of plant-derived extracts and bioactive molecules, and the use of bacteriophages (10). Vaccination has been successful in controlling the disease in livestock in endemic regions (2), but there are no officially approved vaccines for human brucellosis, hindering disease management on a large scale (15). Research on phytomolecules and bacteriophages as alternative therapies for brucellosis is emerging (16–20).

This review discusses the current treatment strategies, mainly antibiotic treatment, the issue of antimicrobial resistance, and alternative therapies for combating brucellosis. The research questions addressed are: (i) what are the reasons behind AMR in brucellosis? (ii) what are the alternative therapies for Brucellosis, and (iii) what strategies can be evolved in developing these alternative therapies to mainstream treatment options. This review was conducted in accordance with the Preferred Reporting Items for Systematic reviews and Meta-Analyses (PRISMA) guidelines to ensure transparency and reproducibility. Literature search was carried out across multiple databases such as PubMed, Web of Science, Elsevier Scopus, and the Directory of Open Access Journals (DOAJ), to identify relevant studies published between 2000 and 2025. However, seminal publications before this time period were included for obtaining a wholesome information. The search strategy utilized a combination of Medical Subject Headings (MeSH) and free-text terms related to brucellosis and alternative therapies. The key MeSH terms included Brucellosis, Brucella, Anti-Bacterial Agents, Antimicrobial Resistance, Bacteriophage, Phage Therapy, Phytochemicals, and Complementary Therapies. Boolean operators (“AND”, “OR”) were employed to refine searches. Only peer-reviewed articles published in English were considered for inclusion. Eligible publications encompassed original research articles and review papers focusing on brucellosis in humans or livestock, antimicrobial resistance, and conventional or alternative therapeutic approaches. Exclusion criteria comprised non-English publications, conference abstracts and non-indexed journals.

The retrieved publications underwent initial screening based on title and abstract, followed by full-text assessment for relevance to the review objectives. Duplicate records were eliminated prior to screening. The shortlisted reports were qualitatively evaluated for study design, clarity of methodology, relevance to brucellosis management, and strength of experimental or clinical evidence. Reports focusing on therapeutic strategies, antimicrobial resistance and mechanism of action of alternative therapies were preferred. The findings were synthesized narratively to outline current challenges and emerging alternative therapies for brucellosis control. Ultimately, the review aims to emphasize the importance of alternative therapies in addressing antimicrobial resistance in brucellosis for both human and livestock.

## 2 Conventional antibiotic treatment

**Figure 1:**
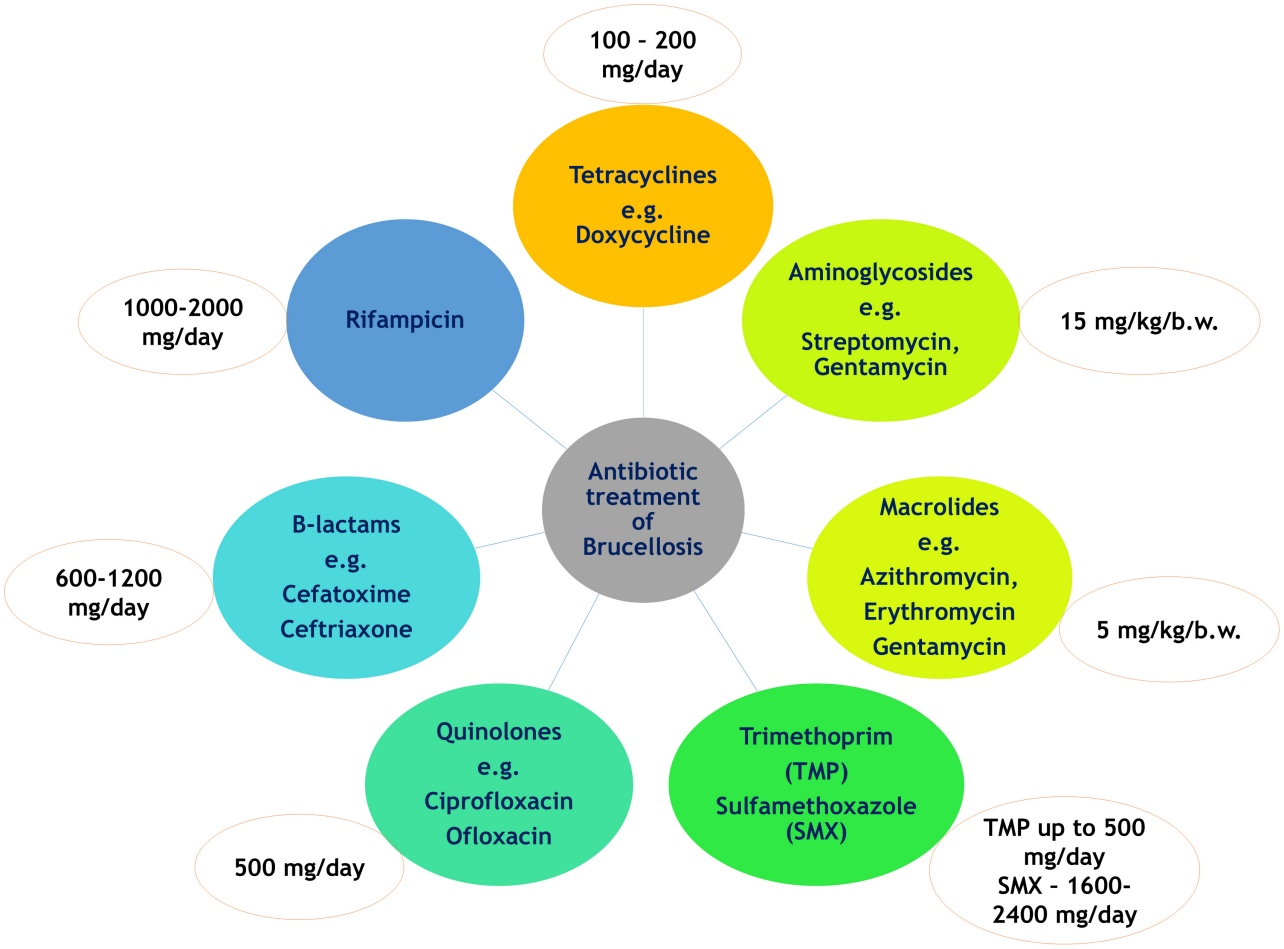
Overview of commonly used antibiotics and their recommended dosages for the treatment of brucellosis.

The key requirements for antimicrobial use in Brucellosis treatment are the ability to penetrate host macrophages, dendritic cells and embryonic trophoblasts and function effectively in the acidic cellular environment (6, 21). The primary objective of antimicrobial treatment for Brucellosis is to reduce symptoms, prevent relapse or chronicity, and decrease *Brucella*-induced mortality. The use of antibiotics in animals is mainly impractical because of the intracellular property of the bacteria in the reproductive organs, lymph nodes, and mammary glands (22). Test and slaughter strategy and vaccination are the main treatment options in case of animals (23). In case of humans, a prolonged course of antibiotic therapy is typically employed in Brucellosis treatment, often using a combination of two or more antibiotics. Various antibiotic classes have been evaluated for human brucellosis treatment, including tetracyclines (e.g., doxycycline), aminoglycosides (e.g., streptomycin), rifampicin, macrolides (e.g., azithromycin and gentamicin), and quinolones (e.g., ciprofloxacin and ofloxacin) (24). A summary of different antibiotics and their recommended dosages in Brucella treatment is provided in Figure 1.

Among the various treatments, inhibitory doxycycline was found to be the most effective antibiotic with excellent activity in the acidic phagolysosomal environment, followed by rifampicin, which possesses excellent cell penetration properties and relatively higher (4–8 folds) anti-*Brucella* activity at acidic pH (25). The minimum concentration (MIC_90_) for doxycycline was 0.004−1 mg L^−1^, while for rifampicin it ranged between 0.02 and 2.5 mg L^−1^ (10). According to the recommendation, a standard procedure of combination therapy consisting of antibiotics from different classes, such as tetracycline and aminoglycosides or tetracyclines with rifampicin, is usually followed (26). This recommendation is based on observations from earlier clinical trials following monotherapy using streptomycin or ceftriaxone (27), ciprofloxacin (28), or trimethoprim and sulfamethoxazole (29), which reported a reduction in symptoms of the disease but with a higher percentage of relapse.

The most commonly used antibiotic regimens for non-complicated brucellosis are: (i) 200 mg oral dose of doxycycline per day for 6 weeks combined with 1 g of streptomycin either through intramuscular (i.m.) or intravenous (i.v.) dosage for the initial 2–3 weeks, (ii) doxycycline dosage as above combined with gentamicin at a dosage of 3–5 mg kg^−1^ day^−1^ provided either through i.m. or i.v. route for 1–2 weeks, and (iii) oral dosage of doxycycline (200 mg) and rifampicin (600–900 mg) per day for 6 weeks (13, 30). Several studies have compared the efficacy of these antibiotic regimens. Treatment with doxycycline + streptomycin for 30 days resulted in faster recovery compared to the doxycycline + rifampicin route (31). A meta-analysis comparing these two regimens involving 544 patients revealed that the streptomycin route resulted in a higher recovery rate (91%) compared to the rifampicin route (82%) (30). However, a comparative study by Acocella et al. (32) revealed no differences between these two standard regimens. On practical grounds, doxycycline combined with rifampicin is a more convenient regimen for outpatient therapy over streptomycin due to the need for parenteral administration, which requires hospitalization during the initial 3 weeks of supplementation with respect to the streptomycin route (33). It has been observed that the combination of quinolones with rifampicin or doxycycline was more efficacious compared to the standard doxycycline + rifampicin treatment. For example, in a randomized clinical study conducted by Hashemi et al., (34), a combination of ofloxacin and rifampicin resulted in a higher cure rate (>93%) with a lower relapse rate (< 8%) compared to the standard doxycycline and rifampicin regimen, which resulted in a cure rate of only 83.9% and a higher relapse rate (15.3%). In another study, multiple combinations were evaluated, such as ciprofloxacin + rifampicin, ciprofloxacin + doxycycline, and doxycycline + rifampicin, as regimens for treating brucellosis (35). The authors observed similar results of cure rate (90%−91%) and relapse rate (7%−8%) with ciprofloxacin + rifampicin and doxycycline + rifampicin, thus identifying newer combinations for standard *Brucella* treatment. However, the treatment regimen and combination of antibiotics vary based on disease severity, availability of the medicine, and age group of the patients (25).

## 3 Antibiotic resistance in Brucellosis

Antimicrobial resistance (AMR) is a significant threat to global health security and has resulted in nearly 4.95 million deaths to date (12). The misuse and overuse of antimicrobials against bacterial infections are the primary drivers behind the development of drug-resistant pathogens. The major mechanisms of AMR include *de novo* mutation and the acquisition of resistance genes from other organisms (36). Common modes of action observed during AMR include inactivation of antibiotics by enzymatic hydrolysis, group transfer, and redox processes, reduced penetration of antibiotics through altered membrane permeability, activation of efflux pumps, and target bypass (37). Bacteria exhibit intrinsic, acquired, or adaptive resistance mechanisms (38). Intrinsic mechanisms involve membrane impermeability to antibiotics in Gram-negative bacteria, while acquired resistance involves mutation and horizontal gene transfer (HGT) through transformation, transduction, and conjugation (39). Adaptive resistance is induced by environmental signals such as growth state, stress, and nutrient availability, and is controlled by epigenetic factors (38). AMR mechanisms mediated through efflux pumps and altered permeability of porins are attributed to adaptive resistance (40).

Brucellosis is endemic in several developing countries and is typically treated with a combination of antibiotics. The antimicrobial treatments are often prolonged and may not effectively inhibit the bacteria due to their intracellular location and sequestration in infected sites such as bones (11). Relapses of the disease are common, with up to 15% occurring in uncomplicated cases, attributed to factors such as failure to eliminate bacteria, inappropriate antibiotic choice, dosage, and duration of therapy (11, 24). The prevalence of other endemic diseases like tuberculosis, which require similar treatment strategies, poses a risk of AMR development (24). While *Brucella* spp. has shown susceptibility to commonly used antibiotics like doxycycline and rifampicin in many studies, resistance has been observed against trimethoprim-sulfamethoxazole and azithromycin in various countries (41–44). However, the phenomenon of AMR has been observed against two classes of antibiotics, viz., trimethoprim-sulfamethoxazole and azithromycin on naturally isolated *B. melitensis* from several multicentre studies. The resistance rates varied widely from 2% in Turkey (45), to 29% in Saudi Arabia both based on broth dilution method (46), 37.5% in India (7), 62% in Saudi Arabia both based on the disc-diffusion method (47), or even 100% in China based on E-method (48) against these antibiotics. In a study by Bosilkovski et al. (13), the authors observed nearly 84% resistance against trimethoprim-sulfamethoxazole when tested in standard *Brucella* broth but high susceptibility when tested in cation-adjusted Mueller Hinton broth (CAMHB). On the contrary, more than 90% resistance was observed for azithromycin in *B. melitensis* in both *Brucella* broth and CAMHB, evaluated through standard microdilution tests recommended by Clinical and Laboratory Standards Institute (CLSI) [https://clsi.org/]. The broth composition and additives supplemented during the cultivation of *Brucella* sp., in the broth and method of testing determine the susceptibility to antibiotics. The presence of certain compounds such as thymine/thymidine reduces the efficacy of sulphonamides such as trimethoprim-sulfamethoxazole (49). This results in generation of false positives making susceptible organisms appear resistant (13). Resistance to monotherapy with antibiotics like streptomycin and ciprofloxacin has been reported in *Brucella melitensis*, with mutations in the rpoB gene implicated in rifampicin resistance in *Brucella* sp., *Mycobacterium tuberculosis* and *E. coli* (50, 51).

Recently, the genetic basis of AMR in *Brucella* sp., was studied from isolates obtained from clinical samples across North East China (52) and India (26) through whole genome sequencing (WGS). A total of 61 isolates were obtained from North East China exhibiting a high degree of resistance to several classes of antibiotics. Approximately 24% strains were resistant to rifampin while nearly 66% and 87% of strains were resistant to cefepime and azithromycin. The authors reported for the first time, cephalosporin-resistant *B. melitensis* in China (52). Apart from this, nearly 28% of isolates were resistant to cefoperazone/sulbactam and < 5% of strains were resistant to cefotaxime (3.3%) and meperidine/sulfamethoxazole (1.6%). The identification of high percentage of cephalosporin resistant (cefepime and cefoperazone/sulbactam) isolates is a significant concern as cephalosporins are very stable and often administered to children, pregnant women and patients having complications of Brucellosis as a first line of treatment [(53) https://pubmed.ncbi.nlm.nih.gov/21378061/]. The higher degree of AMR resistance in Chinese isolates was attributed to the presence efflux pump genes, particularly RND, ABC, and MFS in their genome (52). Efflux pumps play a crucial role in offering AMR to the pathogens. The efflux pumps can be divided into five categories, mainly RND, MFS, ABC, small multidrug resistance family, and multi-antimicrobial and toxic compound extrusion (163).

In the study by Ayoub et al. (26), the genetic basis of AMR was evaluated in *B. melitensis* isolated from India. Twenty-four isolates obtained from infected humans and animals were subjected to whole-genome sequencing (WGS). Sequencing revealed that ~20% of the isolates were resistant to doxycycline, ~16% were resistant to ciprofloxacin and rifampicin and ~4% resistant to cotrimoxazole. The WGS revealed the presence of several AMR genes such as *rpo*B, *fol*P, *gyr*A, *gyr*B, and *par*C, in all the isolates. In addition, the presence of key efflux pump genes, including the multiple peptide resistance factor (*Brucella*_*suis*_*mpr*F) protein and RND-family efflux genes (*bep*C*, bep*D*, bep*E*, bep*F, and *bep*G). Further, the authors identified the presence of *fol*P, a gene known to potentially explain resistance to cotrimoxazole in all isolates. The authors identified nearly 3,000 single nucleotide polymorphisms (SNPs) in the genomes of AMR isolates when compared with the genome *B. melitensis* 16 M. Notably, mutations were observed in the rpoB genes that exhibit resistance to rifampicin (26). Rifampicin functions by binding to the beta subunit of the DNA-dependent RNA polymerase encoded by rpoB gene. Strains resistant to rifampicin (Rifr) exhibit mutations in rpoB gene. Mutations in the rpoB gene lead to overexpression of efflux pumps, increasing resistance to antibiotics (11). A high degree of single nucleotide polymorphism (SNP) has been observed in the use of rpoB gene from clinical isolates of *B. melitensis* from Iran suggesting the use of rpoB gene for differentiation of *Brucella* spp., and their biovars (5). Proteomic profiling has been used to study the mechanisms of AMR, revealing differential expression of proteins in resistant strains, including upregulation of metabolic proteins involved in nucleotide synthesis, energy metabolism, fatty acid biosynthesis, amino acid, transport and binding, cell envelop synthesis (54). The authors observed that proteins such as Na+/H+ antiporters were overexpressed which augment the function of efflux pumps by providing necessary energy for the functioning of drug/multidrug efflux pumps. The authors hypothesized that rifampicin-resistant cells have a more active metabolic profile compared to susceptible cells in order to quickly respond to the stress conditions induced by rifampicin. This response involves the production of cellular components involved in cell wall biosynthesis, efflux of toxic metabolites, and byproducts generated after exposure to rifampicin. Given the prolonged use of antibiotics to treat *Brucella* species and the resulting risk of AMR, there is a need for alternative treatment strategies. Effective vaccination, exploiting plant bioactive compounds and bacteriophage-based therapies offer promising alternatives to combat Brucellosis and reduce the risk of antibiotic resistance.

## 4 Use of herbal extracts for treatment of Brucellosis

Herbal extracts and essential oils derived from aromatic crops have been reported to possess strong antimicrobial properties. These extracts either have intrinsic antimicrobial properties or antibiotic-resistance modifying activities (54). The strong antimicrobial activities of herbal extracts can be attributed to the presence of polyphenols, specifically flavonoids such as isorhamnetin derivatives, quercetin, luteolin, propolin D, catechins, epigallocatechin gallate, and terpenes present in essential oils of various aromatic crops (19). The major mechanisms by which these bioactive molecules function are: (i) cell membrane rupture, (ii) alteration of cell wall permeability, (iii) prevention of bacterial adhesion and biofilm formation, (iv) reduction in fimbriae production, (v) disturbances in protein and DNA metabolism (19). The polyphenols disrupt the membrane structure of microbes by forming pores, altering the electric charge, inducing electrolyte leakage, increasing membrane permeability, modifying membrane fluidity by intercalating with lipid bilayers leading to alteration in biophysical structures (55). Similarly, the terpenes in the essential oils affect the membrane integrity of microbes. The structure of terpenes significantly influences the mechanism of action. For example, certain terpenes such as carvacrol cross the bacterial membrane owing to the presence of polar groups such as phenolic hydroxyl groups and interact with intracellular molecules such as ATP, monovalent cations like K+, and bind to enzymes such as ATPase disrupting cellular homeostasis (55, 56). Other commonly found terpenes such as thymol and cinnamaldehyde incorporate into the lipid bilayer affecting the fluidity of the membrane such as increased rigidity, membrane depolarization ultimately affecting the membrane integrity (57, 58). Furthermore, these terpenes interact with membrane and periplasmic proteins through hydrogen bonding and hydrophobic interactions and alter their biophysical structure (55).

**Figure 2:**
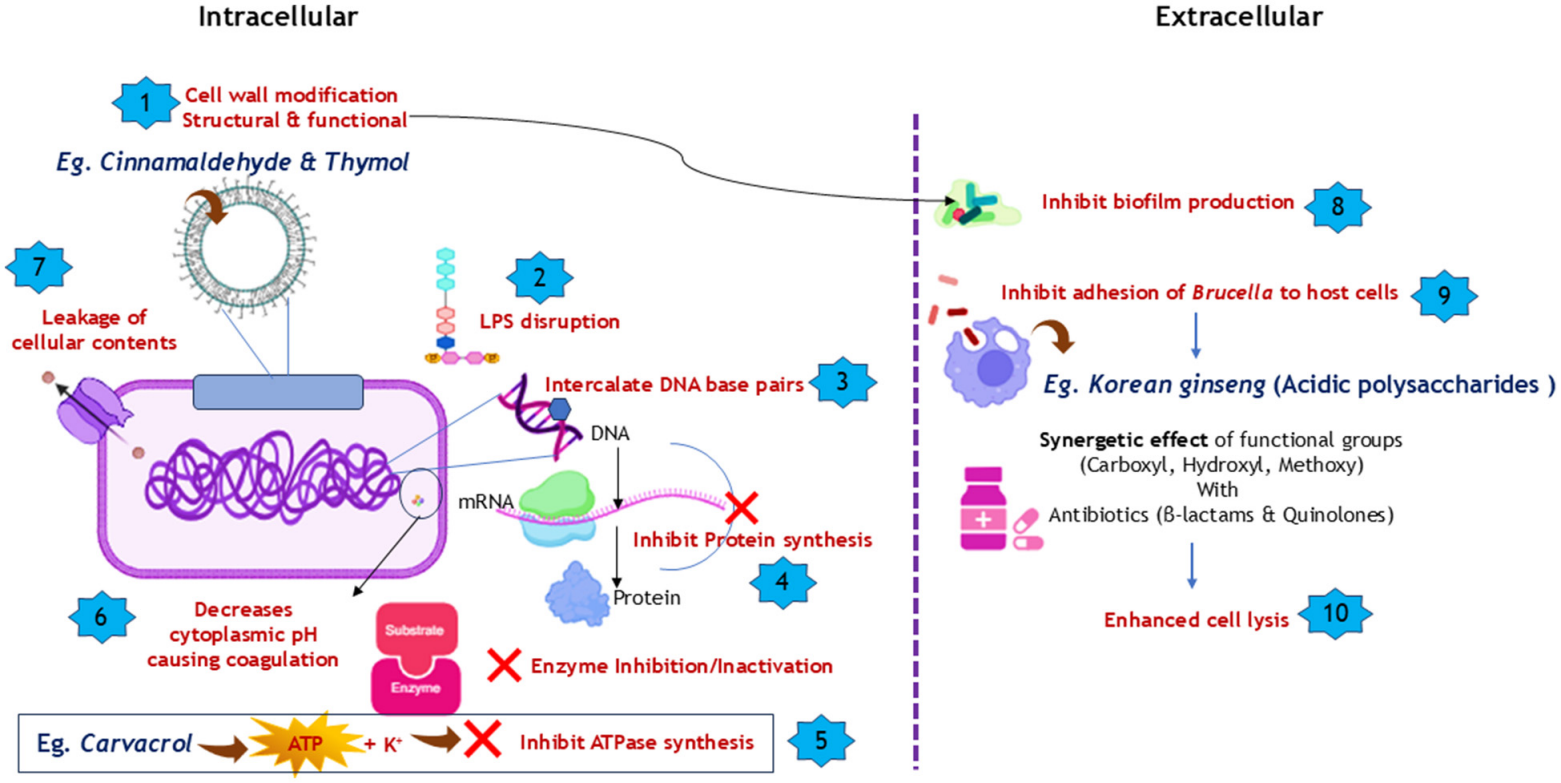
Schematic representation of the proposed anti-brucellar mechanisms of phytochemicals: Phytochemicals exert anti-brucellar effects via membrane and LPS disruption, DNA and protein synthesis inhibition, metabolic interference, biofilm inhibition, reduced adhesion, and cell lysis.

In addition to these aforementioned mechanisms, these bioactive molecules possess the ability to act synergistically with synthetic antibiotics and enhance microbial sensitivity to antibiotics. Polyphenols have been reported to be the predominant class of molecules that act synergistically with synthetic antibiotics (19). Beta-lactams and quinolones are the most commonly utilized antibiotics in evaluating the synergistic effects of plant-derived antimicrobials. The presence of functional groups such as carboxyl (–COOH), hydroxyl (–OH), and methoxy (–O–CH3) in the flavonoids has been attributed to reversing antibiotic resistance (59). The mechanisms behind the synergistic actions are by inhibition of efflux pumps, inhibition of beta-lactamases that degrade antibiotics, and increasing the permeability of bacterial membranes, thus resulting in their damage (60). A schematic representation of the potential mechanisms of actions of different phytomolecules is presented in Figure 2.

Table 1 presents the antimicrobial activity of various classes of phytochemicals against *Brucella* sp. As discussed earlier, the major classes of compounds that exhibit anti-brucellosis activity are phenols and terpenoids. The minimum inhibitory concentrations (MIC) of various plant extracts ranged from 12 to 60 μg ml^−1^ for leaf extracts of *Scrophularia deserti* to 500 mg ml^−1^ for alcoholic extracts of *Alhagi camelorum* Fisch. Alizadeh et al. (16) reviewed the literature on the use of herbal extracts and essential oils with anti-brucellosis activity. So far, 37 medicinal plant species have been reported to possess antimicrobial activity against different strains of *B. melitensis* (Rev1, 16M) and *B. abortus* (S19, S99, 544, and CMCC 210101). In addition to polyphenols and essential oil-derived antimicrobials, polysaccharides from Korean red ginseng (*Panax ginseng* Meyer) have been reported to have anti-*Brucella* activity (61). Acidic polysaccharides from Korean ginseng exhibit immunostimulatory properties, cytotoxic effects against tumors, and anti-inflammatory activity against acute *Staphylococcus aureus* infection (62). These polysaccharides inhibit the adhesion of B. *abortus* cells to host macrophages by inhibiting F-actin polymerization, thereby reducing bacterial invasion into host cells (61). Similar mechanisms of action for ginseng polysaccharides have been observed against other pathogenic bacteria such as *Porphyromonas gingivalis, Actinobacillus actinomycetemcomitans, Propionibacterium acnes*, and *Staphylococcus aureus* (63). Furthermore, the inhibition of F-actin polymerization is achieved through the inhibition of the mitogen-activated protein kinase (MAPK) signaling pathway in *Brucella*-infected macrophages, disrupting cell adhesion. The authors also reported a reduction, rather than inhibition, of intracellular survival of *B. abortus*. This was attributed to the inhibition of phagosome-lysosome fusion in the host cell, followed by the maturation of *Brucella*-containing phagosomes (BCPs) into replicative phagosomes, commonly referred to as brucellosomes (61).

**Table 1:** Antimicrobial activity of herbal extracts against *Brucella* sp.

Name of the medicinal plant	Plant part used	Active ingredient(s)	Inhibitory concentration	Reference
*Alhagi camelorum Fisch*	Aerial parts	Tannins	500 mg ml^−1^	(20)
*Allium sativum*	Cloves	Allicin	1:10 to 1:160	(16, 118)
*Arctium lappa*		Tannins	101–110 mg ml^−1^	
*Berberis integerrima*	Roots	Palmatine Berberine, Columbamine Jatrorrhizine	620 μg ml^−1^ 500 μg ml^−1^ 250 μg ml^−1^ 120 μg ml^−1^	(119)
*Callistemon Citrinus*	Leaf	11-oxo-9-thiocyanato-testosterone	300 mg/ml for *B. abortus, B. melitensis* and 100 mg/ml for *B. suis*	(120)
*Carum carvi*	Seed	Carvone	0.3–2.0 mg ml^−1^	(121)
*Caryopteris mongolica*	Root	Quinone diterpenoids—carnosic acid, carnosol, rosmanol	2.45 mg kg^−1^ body weight	(122)
*Cinnamomum verum*	Bark	Cinnamaldehyde	1% essential oil	(37)
*Cinnamomum zeylanicum*	Bark	Essential oil (Cinnamaldehyde)	MIC_50_ - 3.125 μl/ml and MIC_90_−6.25 μl/ml	(37)
*Citrullus colocynthis*	Aerial parts	Cucurbitacin	25 mg ml^−1^	(3)
*Citrus lemon*	Fruit peels	Coumarin, Tetrazene	1% essential oil	(37, 120)
*Coptis chinensis*	Aerial parts	Berberine	30 mg ml^−1^	(123)
*Cortex phellodendrin*	Stem	Berberine	30 mg ml^−1^	(124)
*Crocus sativus*	Leaf, petals and stigma	Crocin	50–400 mg ml^−1^	(125)
*Cyathula uncinulata*	Leaf, root	Glycoside	1.1 to 2.5 mg ml^−1^	(126, 127)
*Eucalyptus globulus*	Leaf	Cineole	160-166 mg ml^−1^	(128)
*Galla chinensis Sumac*	Root	Gallotannin	30 mg ml^−1^	(129)
*Humulus lupulus*	Flower and cone	Phloroglucinol	0.05–0.625 mg ml^−1^	(130, 131)
*Juniperus oxycedrus L*.	Leaf	α-pinene, limonene and β-pinene	125 μg ml^−1^	(132, 133)
*Korean red Ginseng*	Root	Acidic polysaccharides	0.1–4 mg ml^−1^	(61, 134)
*Lavandula pubescens*	Aerial parts (oval leaves)	Carvacrol, Xanthohumol	10–20 mg ml^−1^	(124)
*Mentha piperata*	Leaf	Menthol	1% essential oil	(37, 135)
*Moringa oleifera*	Leaf, root, seed	Tannins and flavonoids	3-300 mg ml^−1^	(136)
*Myristica fragrans*	Fruit	Myristicin	1% essential oil	(37)
*Nepeta cataria*	Aerial part	Thymol	15.62–125 μl ml^−1^ essential oil	(137)
*Nigella sativa*	Seed	Thymoquinone	1% essential oil	(138, 139)
*Ocimum basilicum*	Aerial part	Linalool	10–40 mg ml^−1^	(140)
*Oliveria decumbens*	Flower	Thymol	50-400 mg ml^−1^	(16, 141)
*Origanum acutidens*	Herbal part	Essential oil—Carvacrol	10 μl	(142)
*Origanum majorana*	Leaf	Terpinen-4-ol	1% essential oil	(37, 135)
*Origanum syriacum*	Aerial parts - Leaf and buds	Carvacol	3.125 μl ml^−1^	(37, 135)
*Peganum harmala*	Seed and leaf	Carboline	50–400 mg ml^−1^	(143)
*Petroselinum crispum*	Seed	Myristicin	0.1–0.2 mg ml^−1^	(144)
*Prunus mahaleb*	Seed	Alpha-Eleostearic acid	1 mg ml^−1^	(144)
*Quercus brantii*	Fruit	Tannins	0.1-0.5 mg ml^−1^	(127)
*Quercus ilex*	Leaf	Catechin	125–500 μl ml^−1^	(145)
*Radix paeoniae Rubra*	Root	Butylidenephthalide	30 mg ml^−1^	(92)
*Salvia sclarea*	Leaf	Linalool	50–400 mg ml^−1^	(125)
*Satureja hortensis*	Leaf	Thymol and carvacrol	31.25–250 μl ml^−1^	(37, 146)
*Scrophularia deserti*	Leaf	Pinene	12–60 μg ml^−1^	(147)
*Teucrium polium*	Aerial parts	Germacrene D	50–400 mg ml^−1^	(125)
*Thymus syriacus*	Aerial parts	Thymol	50 mg ml^−1^	(37, 135)
*Tortille leptophylla*	Fruit	Gallic acid	0.2 mg ml^−1^	(148)
*Vitex pseudo-negundo*	Seed	Hexadecanoic acid (Palmitic acid)	50–400 mg ml^−1^	(149)
*Zataria multiflora*	Aerial parts	Thymol	10–40 mg ml^−1^	(140)
*Ziziphus spina*	Leaf	Linolenic acid	0.1–0.5 mg ml^−1^	(125, 128)

Recently, Zhao et al. (64) conducted a study on the pharmacological activity and signaling mechanisms involved in the adjuvant treatment of Brucellosis using medicinal plants. They employed network pharmacology and bioinformatic analysis to identify five major compounds, primarily polyphenols, including quercetin, kaempferol, luteolin, paeoniflorin, and the phytosterol β-sitosterol. These compounds were found to modulate inflammatory responses and enhance the immune response to *Brucella* infection. Quercetin, in particular, exhibited potent immunomodulatory effects by increasing nitric oxide synthesis and the production of pro-inflammatory cytokines (IL-1, IL1-β, IL-4, IL-6, TNF-α) in response to *Brucella* infection. Additionally, quercetin inhibited the internalization and replication of *Brucella* within macrophages, similar to the mechanism observed with ginseng extracts (61). The potential mechanisms of action of various phytochemicals are listed in Table 2.

**Table 2:** Potential mechanisms of action of various phytochemicals demonstrating antimicrobial activity against Brucella sp.

Mechanism of anti-microbial activity	Class of metabolites	Example	Reference
Cell wall modification (Structural and functional) Cell membrane rupture	•Aromatic aldehyde (flavonoid sub-group) •Monoterpenoid •Flavonoids	•Cinnamaldehyde •Thymol •Epigallocatechin gallate and Quercetin	(19)
Inhibition of efflux pumps leading to anti-microbial resistance (AMR) Binding to outer membrane proteins	•Alkaloids •Monoterpenoid •Cyclic monoterpenes	•Berberine and Palmatine •Thymol •Carvacrol	(55, 150)
Inhibition of biofilm formation Inhibiting of adhesion of Brucella to host macrophages Inhibition of phagocytosis and intracellular replication in macrophages	Acidic polysaccharides from Korean Ginseng Flavonoids	Pectic Polysaccharides composed of Rhamnose (9.5% w/w), Galacturonic acid (18.4%), Galactose (30.4%), Arabinose (35%) Quercetin	(61, 64, 151)
Binding to virulence factors LPS disruption Reduction of bacterial invasion to host	Polyphenols (particularly flavonoids)	Epicatechin gallate, -Morin	(19, 152)
Intercalate DNA base pairs and inhibit DNA binding enzymes (DNA gyrase, DNA helicase) Inhibition of DNA transcription	Polyphenols (particularly flavonoids)	Quercetin, Kaempferol, (+) catechin, Galangin	(19, 151, 153–155)
Inhibition/inactivation of enzymes (ATPase synthesis)	Cyclic monoterpene	Carvacrol	(19, 150, 156)
Intracellular ATP depletion	Aromatic aldehyde (flavonoid sub-group)	Cinnamaldehyde	(157)
Decreases cytoplasmic pH regulation causing coagulation	Phenolic acids Flavonoids	Caffeic acid, Ferulic acid Punigalacin, Quercetin, Galangin	(19)
Membrane rupture and leakage of cellular contents Increased membrane permeabilization	Flavonoids	Quercetin and Kaempferol	(151)
Enhancement of pro-inflammatory cytokines in host cells Inhibiting internalization of Brucella in host macrophages	Flavonoids Monoterpene glucoside Phytosterol	Quercetin, Kaempferol, Luteolin, Paeoniflorin β-sitosterol	(64)

Phytotherapy offers a prophylactic and adjuvant therapeutic options for both human and livestock brucellosis in resource limiting environments where Brucella is endemic. Although theoretically possible, there are no *in vivo* studies demonstrating the efficacy of phytotherapy in Brucellosis treatment. In case of livestock, phytotherapies appear more suitable and scalable under ethno-veterinary practices. Plant based remedies have been widely used to manage symptoms of several livestock diseases including abortions, retained placenta, and for improvement of overall reproductive health across India and African countries like Ethiopia (65, 66). Although, traditionally practiced, a recent scientific report by Kaleem (67) observed that a combination of antibiotics, anti-inflammatory drugs and immunization resulted in better recovery in terms of reduced antibody titres, reduced placenta dropping time and higher birthweight of calves compared to only phytochemical based or only immunization strategies. Although phytotherapy was less effective compared to combination treatment, they still reduced the antibody titer and the Ct values were only marginally lower compared to antibiotic + anti-inflammatory treatment (67). In case of human brucellosis treatments, there are no clinical studies validating the use of phytotherapies. However, herbal remedies have been traditionally used by humans for several microbial infections. Considering the conventional usage, we hypothesize that phytotherapy could be a viable option for treating both human and livestock brucellosis in developing countries owing to its relatively lower costs, accessibility, and the potential to overcome antibiotic resistance.

**Figure 3:**
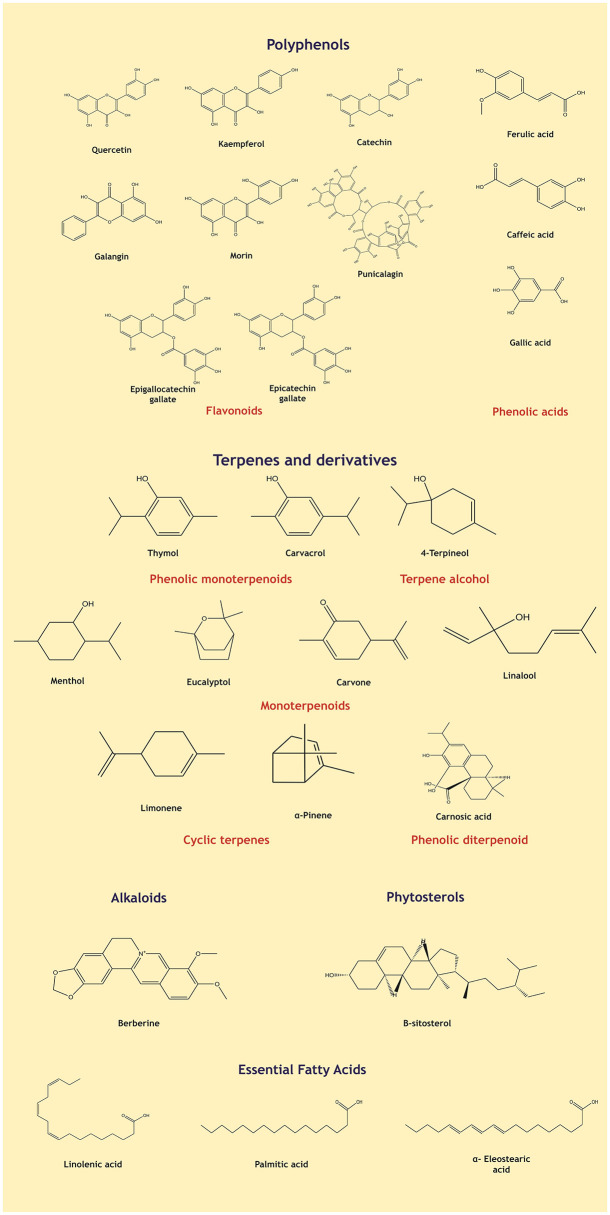
Chemical structures of selected representative phytochemicals reported to possess anti-brucellar activity, categorized into major chemical classes including flavonoids, phenolic acids, terpenes and their derivatives, alkaloids, phytosterols, and essential fatty acids.

Although plant derived bioactive molecules have been reported to possess anti-Brucellar activity, they have certain inherent challenges such as variation in chemical composition owing to biotic and abiotic factors, variations arising to extraction and downstream processes, solubility issues and delivery systems. Further, factors such as durability and compatibility of the phytochemicals with conventional treatments must be evaluated. In addition, selection of the best candidate must involve assessment such as global plant availability and alternative systems of producing the botanical such as cell and tissue culture techniques. Selection fo plants that are scarce or limited to a geography would necessitate captive cultivation of the plants rising the cost of production (68). As evident from Table 1, majority of the phytochemicals exhibiting anti-Brucella activity are either essential oil or enriched fractions consisting multiple compounds acting in synergy. This raises an important challenge of toxicity, as many of these mixtures are yet to be evaluated for systemic toxicity and approved by regulatory agencies such as the U.S. Food and Drug Administration. Misuse of the medicinal -plants are a major concern owing to adulteration, contamination and lack of proper reference standards and guidelines (69, 70). Further development of oral anti-microbials utilizing plant extracts comes with challenges such as bioavailability and stability of phyto-molecules in gastro-intestinal environment (70). In this context, the current research highlights the potential of alternative therapies. The chemical structures of various classes of bioactive molecules reported to exhibit anti-*Brucella*r activity are depicted in Figure 3.

## 5 Bacteriophage therapy for Brucellosis

The widespread prevalence of antimicrobial resistance (AMR) necessitates the development of alternative strategies for treating infectious diseases such as *Brucella*. Bacteriophages offer a biocontrol method for controlling pathogens by inducing cell lysis, making them a potential therapeutic option (71). Bacteriophages exhibit two types of life cycles upon infecting host bacteria: lytic and lysogenic cycles. In the lytic cycle, phages multiply within the bacterial cell and cause lysis to release a new phage population (72). This cycle involves five steps: adsorption, penetration, biosynthesis, assembly, and release of bacteriophages from the infected bacterium. Most virulent phages follow a lytic lifecycle, recognizing specific cell wall receptors such as teichoic acid, lipoteichoic acid in Gram-positive bacteria, and LPS in Gram-negative bacteria (73). Once attached to the cell wall, phages release enzymes to break down the cell wall and insert their genetic material into the host bacterium. Inside the cell, phages synthesize capsid structures, inhibit bacterial replication, and replicate their genetic material, leading to the formation of new phages (74). The genetic material of the phage is transcribed utilizing host transcription machinery to produce mRNA leading to the suppression of host replication. The replication leads to formation of hundreds of phages inside the bacterium. The host cell is lysed when mature phages synthesize holin protein and endolysin to create pores in the cell wall (71).

In the lysogenic cycle, phages integrate their genetic material into the infected bacterium's chromosomes, forming a prophage. Unlike lytic phages, lysogenic phages do not destroy bacterial cells and do not replicate inside them. The integration of phage genetic material can lead to the generation of new bacterial populations. Lysogenic phages, also known as temperate phages, can excise their genetic material under certain environmental conditions such as exposure to UV irradiation, mutagenic antibiotics and cytostatics such as cyclophosphamide destroying bacterial cells (71). In some cases, bacteriophages infect bacteria without destroying them, as seen with filamentous rod-shaped single-stranded phages infecting *Mycoplasma* (75). During excision from bacterial chromosomes, lysogenic phages may incorrectly excise bacterial genes, leading to the generation of new genetic material and facilitating horizontal gene transfer in bacteria. Examples of lysogenic phages include *E. coli* lambda, phage Mu, and ϕ 11 targeting E. coli, *Enterobacteriaceae, Salmonella, Citrobacter*, and *S. aureus*, respectively (76). In addition to their applications in diagnostics, the bacteriophages have various therapeutic applications, including lysing pathogenic bacteria, increasing antibiotic susceptibility in resistant strains, enhancing antibiotic targeting ability and specificity by modulating the genetic makeup of host bacteria (77). Phages can be particularly effective against facultative intracellular bacteria such as *Brucella* sp., *Listeria monocytogenes, Yersinia pestis, Francisella tularensis, Burkholderia pseudomallei, Chlamydia* spp., and *Rickettsia* spp. (78).

The use of bacteriophages for the treatment of brucellosis is a recent development, although their use in the diagnostics of *Brucella* spp., particularly for differentiating *Brucella* biotypes, has been approved by the WHO (26). The phages that target *Brucella* are known as Brucellaphages, which are classified based on their host specificity into Tbilisi (Tb) and Firenze (Fi) that replicate in smooth *Brucella abortus*, and Weybridge (Wb) that replicates in smooth strains of both *B. abortus* and *B. suis* (67). Additionally, new phages like Izatnagar (Iz) have been identified in India, which replicate in smooth *B. melitensis, B. abortus, B. suis*, and some rough *Brucella* strains (79, 80). Recently, another Brucellaphage Palampur (Pr) having ability to infect and lyse multiple various strains of *B. abortus* and *B. melitenis* has been recovered from an aborted buffalo placenta (unpublished work of authors). *Brucellaphage*s belong to the family Podoviridae (67). The main advantage of using phages for therapy is their high specificity and ability to target the pathogenic bacteria without affecting the normal microflora (81). Phages replicate until the pathogenic bacteria are eliminated, making a single administration of a specific bacteriophage sufficient for treatment. In the absence of the host bacterium, phages are inert, non-living protein coats containing genetic material (82).

The efficacy of phage treatment in controlling *Brucella* was demonstrated in a mice model infected with *B. abortus* 544 strains, where the phages lysed the pathogen and reduced *Brucella* colonization in the host (83). Furthermore, *Brucella* phage lysates of *B. abortus* S19 strain were effective in inducing antibody production and cell-mediated immunity in mice challenged with the pathogen (*B. abortus* 544 strain) (84). In an experimental trial, guinea pigs immunized with *Brucella* S19 phage lysate showed significant IgG antibody production. Transfer of these antibodies to rats provided passive protection against the pathogen (85). Mohan and Saxena (18) studied the effect of phage therapy in *Brucella*-infected cattle and observed a significant immune response that declined after 6–7 weeks. However, when the lysates were administered with live attenuated *B. abortus* S19 vaccine, the cattle produced high titers of anti-*Brucella* antibodies, with sustained immune response and clearance of intracellular *Brucella* (86). Different phage lysates induce varying immune responses, with smooth *B. abortus* S19 strain lysates inducing IgG antibody production in cattle and rough *B. abortus* RB 51 strain lysates inducing cell-mediated immunity. A combination of both lysates injected subcutaneously in cattle effectively removed live bacteria from infected cattle (87). However, use of lysogenic phage involves certain risks such as triggering of immune cascade in host cells. For example, phage induced Brucella lysate can induce exaggerated immune response in host. This is because, rapid lysis of Brucella could lead to the release of large quantities of antigens, particularly Pathogen Associated Molecular Patterns (PAMPs) leading to pro-inflammatory cytokine storm. For example, Brucella lipopolysaccharides (LPS) bind to TLRs that induce strong pro-inflammatory cytokines release. Such excessive inflammatory triggers can lead to systemic shock in the host (88, 112). Duan et al. (88) reported release of large quantities of LPS after phage lysis of multidrug-resistant *Serratia marcescens* in host cells leading to stimulation of immune response resulting in the elevated levels of IL-6 and C-reactive protein (CRP).

**Figure 4:**
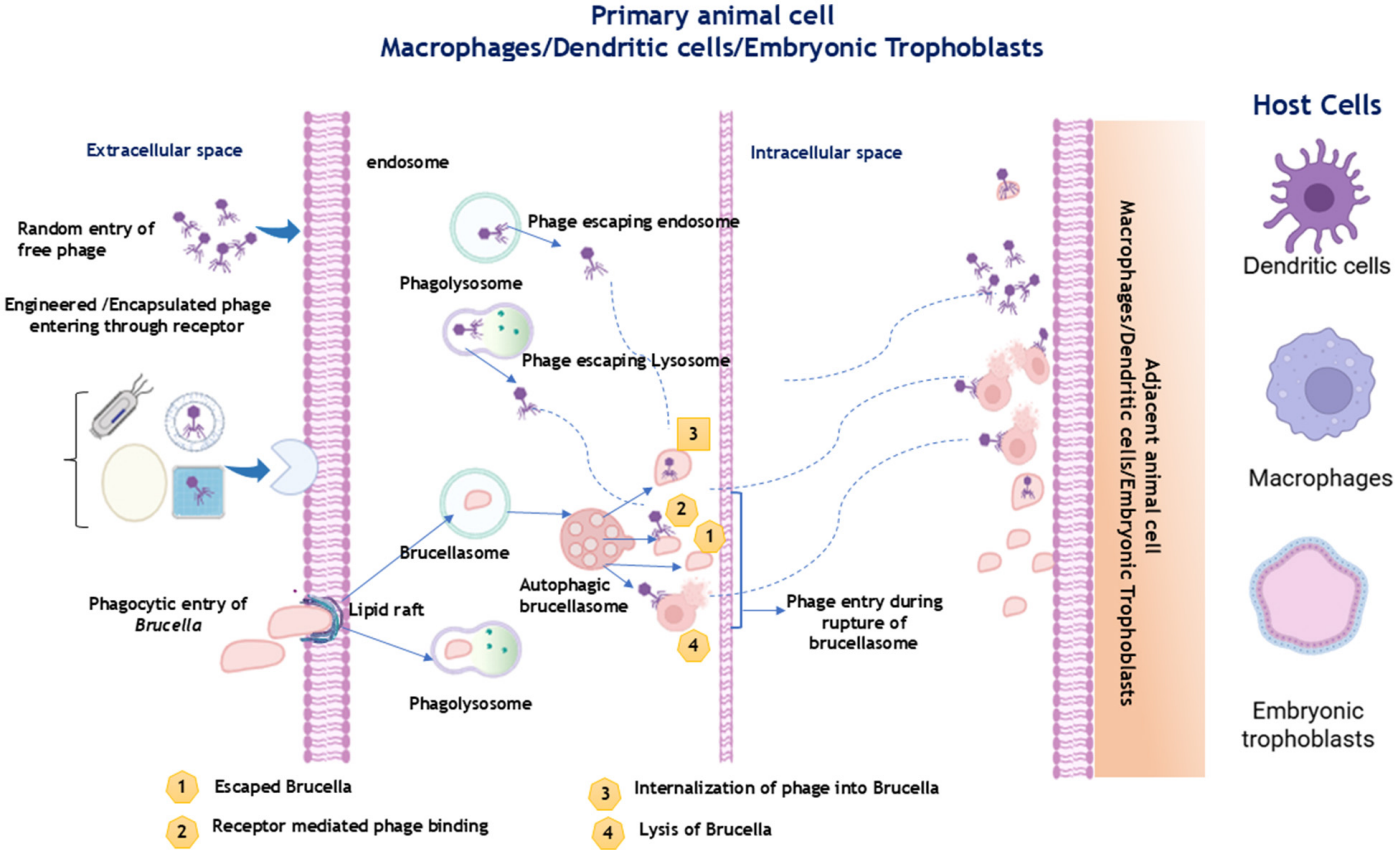
Schematic representation of Brucellaphage–host cell interactions consisting Brucellaphage entry, intracellular trafficking, interaction with Brucella-containing vacuoles, bacterial lysis, and phage dissemination to adjacent host cells (Images created using Biorender).

Phages are typically sourced from sewage, litter material, soil, and fecal pits in cattle farms (20). Recent studies by Mohan and Saxena (18, 86); Shaheen et al. (89) and Ötkün et al. (20) have demonstrated the efficacy of bacteriophages in controlling various *Brucella* species, highlighting the potential of phage therapy as a viable treatment option for bovine brucellosis. The potential mechanism and a schematic representation of interaction between Brucella and bacteriophage is presented in Figure 4. The interaction between bacteriophages and *Brucella* involves receptor-mediated binding, internalization, and lysis of *Brucella* cells, leading to a robust immunological response compared to live attenuated vaccines. The host specificity and potential applications of Brucellaphages are summarized in Table 3.

**Table 3:** The host specificity and potential applications of Brucellaphages.

Application	Description	Examples	References
Biotyping/Diagnosis	Identifying and differentiating *Brucella* strains based on phage sensitivity	Tbilisi (Tb) phage for typing smooth *Brucella abortus*	(158)
Therapy	Treating active *Brucella* infections in livestock	Phage cocktails in animal models	(67, 159)
Biocontrol	Using phages to control *Brucella* in the environment or animal hosts	Tbilisi (Tb), Weybridge (Wb), Firenze (Fi) phages	(160, 161)
Prophylaxis (Vaccine Development)	Using phages to protect against *Brucella* infection in livestock	Research ongoing	(162)

## 6 Recent trends and future prospects in *Brucella* therapy

Given the recent rise in antimicrobial resistance (AMR) in *Brucella* species, there is a pressing need for the identification of alternative strategies. Live-attenuated vaccines have historically been effective in controlling the disease. However, their drawbacks, such as interference with serodiagnostic assays, virulence in humans, and antibiotic resistance, highlight the necessity for the development of novel immunization strategies (90). Subunit vaccines and genetically engineered vaccines have shown efficacy in inducing immune responses in animals challenged with the pathogen. Vaccine strains with deletions of genes involved in virulence (T4SS, lipid A fatty acid transporting gene involved in LPS biosynthesis) and cell survival (ferrochelatase hem H, phosphoglycerate kinase, purine biosynthesis) have been effective in inducing cell-mediated immunity in hosts (91). Additionally, the use of subunit vaccines containing important virulence factors such as LPS, OMP, and Bscp31 has been found to elicit specific antibodies against the pathogen (92, 93). Heterologous production of virulence factors in attenuated vectors such as *Salmonella* sp., *Influenza* virus, or Adenovirus can provide highly specific immune responses in hosts and significant therapeutic potential (94, 164). Recent advancements in vaccine production include the use of nanoparticle-based oral vaccines and recombinant vaccines containing *Brucella* antigens. Conjugation of OPS and LPS antigens from *B. abortus* to nanoparticles has offered significant protection against *Brucella* species (95). Nanoparticle vaccines elicit Th1-mediated immunological responses and triggers the production of IgM, IgG, and IgA antibodies (96). While nanoparticle delivery provides protection against the pathogen and offers an easier oral route option, the technology carries risks due to toxicity, limitations in antigen loading, and suboptimal immunological responses (3). Nevertheless, vaccines developed using novel technologies that utilize recombinant *Brucella* proteins and nanoparticles hold promise for future use in preventing this zoonotic disease.

**Figure 5:**
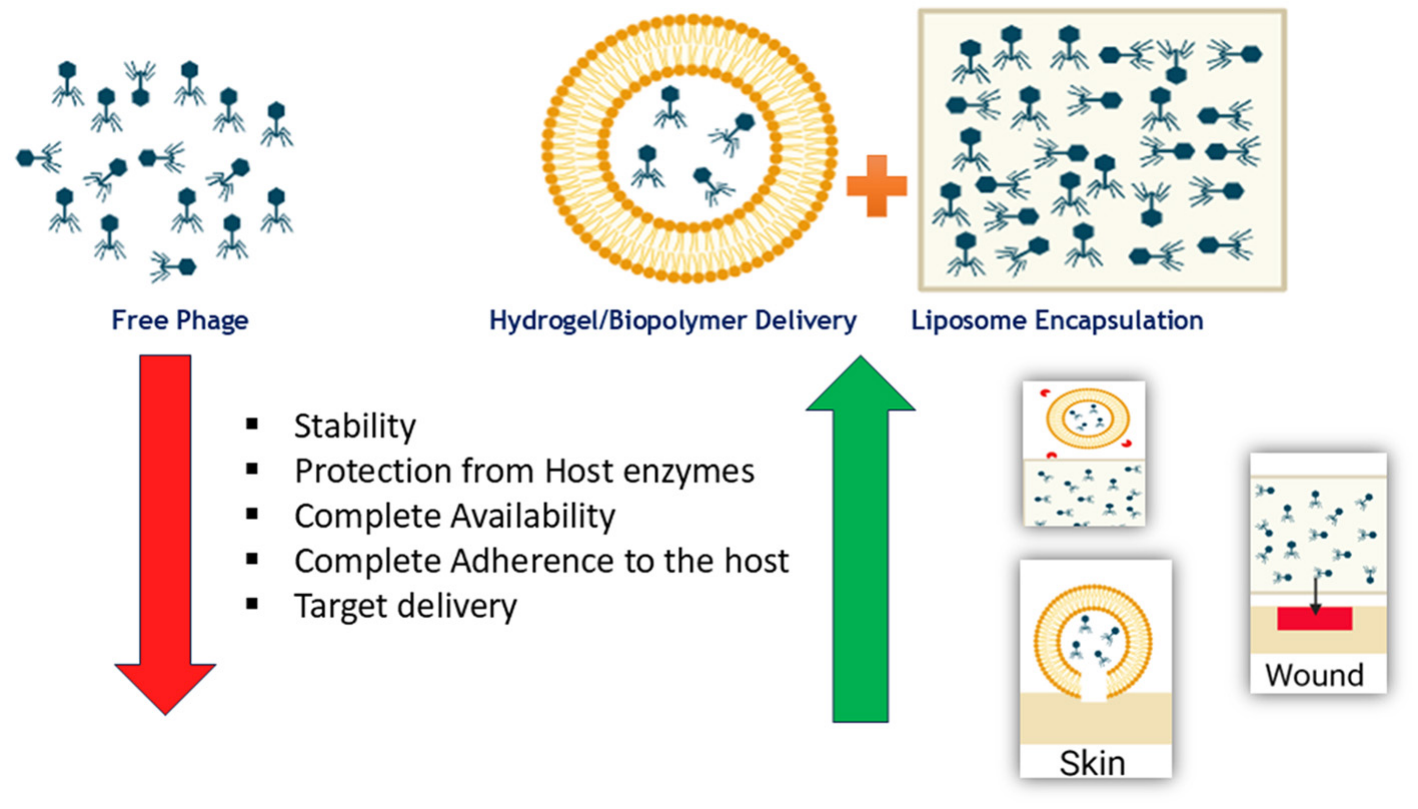
Advantages of phage encapsulation over free phage delivery in host systems. Encapsulation using liposomes or hydrogel/biopolymer matrices enhances phage stability, protects against degradation, improves targeted delivery and retention at infection sites (skin and wounds), and enables controlled release, resulting in improved therapeutic efficacy [modified from Dürr and Leipzig (77)].

Plant-derived bioactive molecules have long been used as antimicrobial agents, but their extractability and poor solubility in aqueous media are major limitations. Conjugating these bioactive molecules with water-soluble moieties or nanoencapsulating the extracts can enhance their solubility and bioactivity (19). Recent studies on the anti-Brucellar activity of plant-derived polyphenols (quercetin, kaempferol, luteolin, paeoniflorin) and a phytosterol (β-sitosterol) have shown their ability to improve the immune response to *Brucella* infection, offering novel adjuvant therapy options for controlling brucellosis (64). However, systematic studies on large populations are necessary to validate their efficacy. In this context, phage therapy presents a viable solution for controlling brucellosis and increasing sensitivity to antibiotics in resistant strains. While phage therapy has shown success, it faces inherent challenges related to stability and efficacy. Delivering free phages to host organisms infected with pathogenic bacteria results in lower half-life due to gastric enzymes, poor adherence to bacterial biofilms, and other production-related stresses (97). Encapsulating phages using appropriate biopolymers such as liposomes, hydrogels, or nanovesicles enhances their stability by limiting exposure to extreme pH and degrading enzymes. Hydrogel encapsulation offers localized delivery of phages to infected/wound sites, increasing phage titers and adherence to the site, thereby improving therapy efficacy. Liposome-mediated phage delivery has been effective in enhancing phage adhesion and interaction with the target site (77). Biopolymers evaluated for phage delivery include hydrogels derived from proteins such as collagen and fibrin, polysaccharides such as agarose and alginates, and synthetic polymers like polyethylene glycol (PEG), polyacrylamide (PA), and polyvinyl alcohol (PVA) as wall materials for phage encapsulation (98). Nano-encapsulation techniques enable controlled delivery of phages triggered by specific cues such as environmental fluctuations, temperature, pH, and exposure to hydrolytic enzymes (99). The benefits of phage encapsulation over free phage delivery in the host system are illustrated in Figure 5.

### 6.1 Phage engineering as a novel-strategy for diagnosis and control of *Brucella*

Phages exhibit remarkable specificity toward their bacterial hosts, making them valuable tools for diagnostic applications. They can be used as bio-probes in conjunction with radioactive tracers, fluorophores, and nanoparticles to detect bacterial strains (100). The specificity of bacteriophages is mediated by receptor binding proteins (RBPs), which can be isolated and synthesized in different heterologous expression systems for use in ELISA-based assays. For instance, Denyes et al. (101) utilized engineered Bacteriophage S16 tail fiber proteins for detecting *Salmonella* cells. In the case of Brucellaphages, specific endolysin proteins binding to *Brucella* cell wall proteins, such as LPS, OMPs, and OMV proteins, can be identified and engineered for accurate detection of *Brucella* strains. Alternatively, phage infection-based detection methods can be employed. Bacterial strains can be infected with lytic phages, and the resulting lysate contains specific bacterial cytosolic proteins can serve as markers for detection (102, 103).

Furthermore, phages can be engineered with genes encoding reporter proteins, such as fluorescent protein luciferase or hydrolyzing enzymes like β-galactosidase. These proteins are amplified during phage replication inside cells and can be detected by adding appropriate substrates (104, 105). These phage-reporter systems are capable of detecting very low concentrations of bacterial cells, particularly *Brucella*, which have low circulating concentrations in blood.

Engineered phages can be produced using various techniques, such as homologous recombination, phage cross technique, or CRISPR-Cas9 system. The CRISPR-Cas9 system can enhance the lytic activity of phages and generate more potent antibacterial agents by introducing recombinant genes into the phage DNA (106). By modifying the specificity of phages, they can be tailored to target a broader spectrum of hosts or focus on specific species or variants, which is beneficial for treating multi-drug-resistant bacterial strains (107). In the context of *Brucella*, genetic engineering can be utilized to develop phages specific to different biovars, and broad-spectrum phages can be used to combat diverse bacterial infections (108).

Tailoring the specificity of bacteriophages can reduce off-target effects on beneficial microflora in animals and humans (109). Additionally, genetic engineering can be employed to increase the biological half-life of phages, evade immune detection, or dampen immune responses (107). Bacteria can develop resistance to phages through various mechanisms such as (i) preventing the adsorption of phages to their cell wall, (ii) prevention of phage entry, (iii) targeted cleavage of phage nucleic acids, (iv) abortive infections and (v) prevention of prophage formation (110). These resistance mechanisms can be overcome by engineering phages to evade bacterial defenses. Phage cocktails can also be used to broaden the spectrum of treatment and prevent the development of phage-resistant bacterial mutants (111).

While phages have been successful as therapeutic agents, their efficacy against intracellular pathogens like *Mycobacterium* sp. and *Brucella* sp. is limited due to their intracellular nature (112). Genetic engineering strategies can be employed to enhance the effectiveness of phages against such pathogens. Phages can be engineered to display mammalian cell surface receptors or ligands to facilitate their entry into mammalian cells. Engineering capsid proteins to interact with mammalian cell membranes can improve their ability to penetrate cells, and rational design or directed evolution approaches can optimize capsid structure for mammalian cell penetration (113, 114).

### 6.2 Prophylactic use of phages in control of Brucellosis

In addition to their diagnostic and therapeutic uses, phages can be effectively employed for the prophylactic treatment of Brucellosis. In this approach, stable and environmentally resistant phage formulations in the form of aerosol sprays can be utilized for the biocontrol of pathogenic bacteria. For *Brucella* species, these prophylactic phage formulations can be beneficially applied in cattle farms and meat processing facilities. However, key challenges include selecting stable phages, developing practical methods of phage administration, and formulating products that maintain phage viability (115). Alternatively, phage lysozyme proteins such as endolysin and phage tail-associated murein lytic enzymes (TAME) can be used prophylactically to hydrolyze bacterial cell walls. These proteins have been utilized as additives to control microbial contamination during controlled fermentation in dairy and brewery industries. Phage endolysins have demonstrated efficacy in preventing contamination of fermented products by a wide range of Gram-positive bacteria (116). Specific phage endolysins targeting different *Brucella* strains can be produced using recombinant technologies and employed as prophylactic agents. The stability of phages can be enhanced through nanoencapsulation techniques such as liposome or biopolymer encapsulation, as discussed in the previous section. Alternatively, feed and water consumed by *Brucella*-susceptible cattle could be treated with specific bacteriophages. The food industry has successfully utilized phages and phage-derived proteins for microbial decontamination of products, with minimal impact on food quality (116, 117). Overall, the literature strongly supports the potential applications of bacteriophages as diagnostic, therapeutic, and prophylactic agents in the treatment of livestock brucellosis.

## 7 Conclusion

*Brucella* is a major zoonotic disease causing significant economic losses in resource-limited countries. Due to its intracellular nature and stealthy pathogenesis mechanism, *Brucella* evades the host immune system and requires prolonged antibiotic treatment. However, there is a high relapse rate and increasing resistance to commonly used antibiotics particularly cephalosporins, azithromycin, rifampicin in recent years, necessitating the identification of alternative treatment strategies. Live attenuated vaccines have been traditionally used in immunization programs for livestock and have been successful in eradicating the disease in certain regions. However, drawbacks such as virulence to humans, interference during sero-diagnosis, and a high rate of abortion in cattle have led to the development of alternative vaccine strategies. The use of plant-derived bioactive molecules as adjuvants to conventional therapy has shown success in recent trials. Plant polyphenols and terpenoids could be targeted as a source of novel antimicrobials against *Brucella* in light of antimicrobial resistance. Phytochemicals function similar to antibiotics by disrupting cell membrane, prevent adhesion of Brucella sp., to host cells, inhibit virulence factors and inhibit DNA replication and transcription and enzyme function. Phytotherapies can be explored as adjuvant treatment options in both livestock and human brucellosis. Although effective, caution is warranted when extrapolating *in vitro* results to *in vivo* clinical applications of the phytotherapies. Bacteriophage mediated strategies could be explored for control of Brucellosis through different applications viz., biotyping and diagnosis of *Brucella* sp., therapeutic applications for lysis of *Brucella* sp., particularly in livestock, and as biocontrol/prophylactic agent in control of *Brucella* in the environment and host. The challenges associated with delivery of phages to infected site can be addressed through nano-encapsulation techniques utilizing biopolymers. Further the specificity and efficacy of phage action on *Brucella* sp., can be enhanced through genetic engineering strategies. Additionally, genetic engineering can be employed to increase the biological half-life of phages, evade immune detection, or dampen immune responses in host. In conclusion, plant-derived bioactive molecules could be used as prophylactic and adjuvant treatment strategies for both livestock and humans, while bacteriophages could serve as therapeutic agents for the control of brucellosis, particularly in livestock.
